# The pathology of oxidative stress-induced autophagy in a chronic rotator cuff enthesis tear

**DOI:** 10.3389/fphys.2023.1222099

**Published:** 2023-09-11

**Authors:** Renaldi Prasetia, Siti Zainab Bani Purwana, Ronny Lesmana, Herry Herman, Bancha Chernchujit, Hermawan Nagar Rasyid

**Affiliations:** ^1^ Department of Orthopaedics—Traumatology, Hasan-Sadikin General Hospital, Universitas Padjadjaran, Bandung, Indonesia; ^2^ Faculty of Medicine, Hasan-Sadikin General Hospital, Universitas Padjadjaran, Bandung, Indonesia; ^3^ Department of Biomedical Sciences, Division of Physiology, Faculty of Medicine, Universitas Padjadjaran, Bandung, Indonesia; ^4^ Department of Orthopaedics Surgery, Faculty of Medicine, Thammasat University, Rangsit, Thailand

**Keywords:** rotator cuff tear, enthesis healing, chronicity, oxidative stress, autophagy

## Abstract

Partial-thickness rotator cuff tears (PTRCTs) are often found in daily orthopedic practice, with most of the tears occurring in middle-aged patients. An anaerobic process and imbalanced oxygenation have been observed in PTRCTs, resulting in oxidative stress. Studies have shown the roles of oxidative stress in autophagy and the potential of unregulated mechanisms causing disturbance in soft tissue healing. This article aims to review literature works and summarize the potential pathology of oxidative stress and unregulated autophagy in the rotator cuff enthesis correlated with chronicity. We collected and reviewed the literature using appropriate keywords, in addition to the manually retrieved literature. Autophagy is a normal mechanism of tissue repair or conversion to energy needed for the repair of rotator cuff tears. However, excessive mechanisms will degenerate the tendon, resulting in an abnormal state. Chronic overloading of the enthesis in PTRCTs and the hypovascular nature of the proximal tendon insertion will lead to hypoxia. The hypoxia state results in oxidative stress. An autophagy mechanism is induced in hypoxia via hypoxia-inducible factors (HIFs) 1/Bcl-2 adenovirus E1B 19-kDa interacting protein (BNIP) 3, releasing beclin-1, which results in autophagy induction. Reactive oxygen species (ROS) accumulation would induce autophagy as the regulator of cell oxidation. Oxidative stress will also remove the mammalian target of rapamycin (mTOR) from the induction complex, causing phosphorylation and initiating autophagy. Hypoxia and endoplasmic reticulum (ER) stress would initiate unfolded protein response (UPR) through protein kinase RNA-like ER kinase (PERK) and activate transcription factor 4, which induces autophagy. Oxidative stress occurring in the hypovascularized chronic rotator cuff tear due to hypoxia and ROS accumulation would result in unregulated autophagy directly or autophagy mediated by HIF-1, mTOR, and UPR. These mechanisms would disrupt enthesis healing.

## 1 Introduction

A rotator cuff tear is a common shoulder soft tissue injury in adults (Eljabu et al., 2015; Nakamura et al., 2015; Kwong et al., 2019). The incidence of tear repair failure is high (ranging from 30% to 94%), despite the advancements in surgical techniques (Nakamura et al., 2015; Chalmers et al., 2016; Prabhath et al., 2021). Inadequate response to tissue healing in the enthesis could be the cause of this problem (Prabhath et al., 2021). The prevalence rate of partial-thickness rotator cuff tears (PTRCTs) ranges from 13% to 32%, which is as common as full-thickness rotator cuff tears (FTRCTs) (Tsuchiya et al., 2021). PTRCTs are often found in daily orthopedic practice, with most of the tears occurring in elderly patients (Eljabu et al., 2015). The known natural history of a rotator cuff tear described in the literature is still lacking. Understanding of the nature of this injury could be helpful in the application of investigations, follow-ups, treatment determination, and complication prevention. Further knowledge of the injury, healing, and regeneration mechanisms of a rotator cuff tear is needed to elaborate the causes of the recurrent tear (Keener et al., 2015; Chalmers et al., 2016; Tsuchiya et al., 2021).

In a rotator cuff tear, tear progression would occur, creating a larger tear (>2–5 mm increase in size) or transforming a partial tear to a full-thickness tear (28–41.53%) (Eljabu et al., 2015; Nakamura et al., 2015; Kwong et al., 2019; Oh et al., 2020; Ichinose et al., 2021; Tsuchiya et al., 2021). Nakamura et al. (2015) observed tear progression by examining the initial localization and then comparing it to the location of the spread and found that 22.8% of the injury spreads posteriorly or anteriorly. PTRCTs have less risk of tear progression at 8.4–44% in 6–100 months and 0.26% progression per month compared to FTRCTs at 37.4–82.4% in 6–100 months and 0.85%–1.00% progression per month (Keener et al., 2015; Kim et al., 2017; Kwong et al., 2019; Tsuchiya et al., 2021). With the aforementioned progression rate, we still have to consider the possibility of an increase in the rate when tears reach a certain size (Nakamura et al., 2015). Keener et al. (2015) showed that a tear in the dominant shoulder had greater tear enlargement (63%) than a tear in the non-dominant side (42%), indicating the influence of the activity level. Another mechanical mechanism of tear progression is mechanical impingement caused by a subacromial spur, but this is mainly observed in a tear located at the anterior part of the superior facet (Nakamura et al., 2015). Biological causes, such as degeneration, still play an important role in tear progression (Nakamura et al., 2015; Hebert-Davies et al., 2017). Conservative treatment is often used for an asymptomatic tear; however, asymptomatic and symptomatic tears had no significant differences in the progression rate (Nakamura et al., 2015; Tsuchiya et al., 2021). This enlargement would decrease shoulder function significantly compared to the shoulder function in the initial tear state (Keener et al., 2015). The progression into a greater tear not only affects tear severity in patients but also contributes to pain development, inducing new pain from asymptomatic tears or increasing the visual analog score by an average of 3 points, contributing to the decline in shoulder function (Eljabu et al., 2015; Keener et al., 2015; Keener et al., 2019). The pain felt by patients could be due to a few pathology mechanisms such as impingement and inflammation (Ichinose et al., 2021). Tendon healing after surgery is decreased in an enlarged tear (Keener et al., 2015).

Problems arising in healing failure could be caused by factors such as the technique used for the repair, early mechanical failure from an unsound repair, and biological healing failure. Rotator cuff tear repair with tissue preservation had shown a greater re-tear rate than repair with bursal tissue detachment. Pathological tissues not successfully removed in the repair will be retained, often the cause of recurrent pain (Prasetia et al., 2021). Deniz et al. (2014) showed that fatty degeneration and atrophy occurring before repair remain unresolved even after repair treatment. These factors arise as a prominent problem in healing, and pathological processes need to be considered to solve those (Prasetia et al., 2021).

An anaerobic process and imbalanced oxygenation have been observed in PTRCTs, resulting in oxidative stress (Lui et al., 2022). The induction of cascades by build-up and prolonged oxidative stress could result in unwanted unregulated mechanisms occurring in the chronically torn enthesis (Hirunsai and Srikuea, 2021; Lui et al., 2022). Studies have elucidated the roles of oxidative stress in autophagy and the potential of unregulated mechanisms causing disturbance in soft tissue healing, with autophagocytic pathways that remodel and degenerate the tissue to adapt to the chronic rotator cuff tear (Gumucio et al., 2012; Hirunsai and Srikuea, 2021). Tissue degeneration can be grossly and histologically observed in the chronic rotator cuff tears as denervation degeneration or atrophy and fatty infiltration (Ditsios et al., 2014; Zheng et al., 2019). Autophagy in degeneration had been discussed in the liver, nervous system, and muscular disorders but rarely mentioned in tendon injury, especially in an enthesis tear, with chronicity worsening the degeneration (Wu et al., 2011). This article aims to review the literature works and to summarize the pathology of oxidative stress and unregulated autophagy in the rotator cuff enthesis correlated with chronicity. We aim to provide a clear mechanism potentially involved in the delayed enthesis healing of a chronic rotator cuff tear so that this review could be used as a reference for further study to improve healing through nonoperative or operative management. Literature works were retrieved from online resources, including PubMed, ScienceDirect, and Scopus, using appropriate keywords, in addition to the manually retrieved literature.

## 2 Mechanisms involved in a rotator cuff tear

### 2.1 Chronicity of a rotator cuff tear

Time-dependent risks of tear enlargement have been demonstrated in the literature (Hebert-Davies et al., 2017; Codding and Keener, 2018). Patients with a rotator cuff tear left untreated for more than 24 months had a higher re-tear rate (20%) than patients treated early with surgery (13%) (Codding and Keener, 2018). Cuff pathology would alter tissue quality and would impair tissue healing (Sambandam et al., 2015). A chronic rotator cuff tear will progress and degenerate, the reason why surgical repair is considered the early management in the course of the disease (Eljabu et al., 2015; Nakamura et al., 2015). The progression of a rotator cuff tear has the potential to increase the risk of tendon retraction, muscle atrophy, and fatty infiltration (Eljabu et al., 2015; Tsuchiya et al., 2021). In the investigation, symptom progression can be clinically observed as a possible sign of tear enlargement. Arthrography, ultrasound, and magnetic resonance imaging are used to evaluate tear progression and degeneration (Keener et al., 2015; Nakamura et al., 2015).

With greater progression involved in a chronic rotator cuff tear, greater kinematic tension is altered. When the tear achieves an 175 mm^2^ area threshold, the proximal humerus starts to migrate, producing further tension, mechanically pulling the tear, which contributes to tear progression (Codding and Keener, 2018). Muscle atrophy occurs as a complication of a chronic rotator cuff tear, which blunts the muscle capacity to produce force (Zheng et al., 2019). The muscle force of a chronic cuff tear had been studied in rats by Ditsios et al. (2014). The muscle strength showed a significant reduction by 30% and 35% in muscle force in the supraspinatus muscle and infraspinatus muscle, respectively (Ditsios et al., 2014). The impaired tension and chronic overloading would eventually lead to degeneration, where soft tissues underwent fatty infiltration and atrophy (Keener et al., 2019). The angulation between muscle fibers is altered by retraction, allowing adhesions to occur. Loading alterations change the muscle structure, which may be induced by adhesions (Sambandam et al., 2015).

The pathology involved in a tear would eventually contribute to fatty infiltration, increased connective tissue content, and fibrosis, decreasing the elasticity, viability, and healing of the cuff (Sambandam et al., 2015). Fatty degeneration is significantly found more often in a symptomatic tear (35% vs*.* 4%), but supraspinatus muscle atrophy is found in both symptomatic (35%) and asymptomatic (12%) rotator cuff tears as shown in Kim et al. (2017). Degeneration is influenced by age, as observed in many older patients (Narvani et al., 2020). Ditsios et al. (2014) studied the chronic rotator cuff tear in animal models by detaching rats’ supraspinatus and infraspinatus muscles in a procedure and harvesting the results after 12 weeks and showed the histological characteristics. Fibrosis and adipose tissue proliferation in the endomysial tissue led to degeneration with muscle fiber separation and the loss of its original polygonal shape. Atrophic fibers surrounded by normal or hypertrophic fibers were also found, indicating denervation-type degeneration (Ditsios et al., 2014). Necrosis was also prominent, indicating the possibility of excessive protein degradation (Ditsios et al., 2014; Zheng et al., 2019).

It has been shown that progressive degenerative changes and tear enlargement have a significant association (Keener et al., 2015; Hebert-Davies et al., 2017). Keener et al. (2015) found that a stable tear had, respectively, 4% and 9% supraspinatus and infraspinatus degeneration, which increased to 30% and 28% degeneration in shoulders with tear enlargement. Tears with degenerative transformation had a higher chance of progression (79% vs*.* 58%) according to Hebert-Davies et al. (2017). Muscle degeneration was observed more frequently in a progressive tear with a 15.0 mm baseline size. Progressive tissue degeneration involved in a tear lowers the tendon healing rate and renders irreparability after surgical management, and although it does not happen to all cases, this could lead to shoulder disability (Eljabu et al., 2015; Keener et al., 2015; Nakamura et al., 2015). Other studies have also correlated the increase in the rotator cuff tear size with fatty muscle infiltration (Hebert-Davies et al., 2017). The increasing size of a tear with fatty degeneration may be irreparable in time (Narvani et al., 2020). Further degeneration can complicate the tear into rotator cuff tear arthropathy with proximal humeral migration, acromial acetabularization, glenohumeral chondral loss, and bone loss (Chalmers et al., 2016).

Rotator cuff tears mostly occur in the bicep tendon (13–17 mm posterior). This area is the rotator crescent, with the rotator cable as its border, and it correlates with the junction of the infraspinatus and supraspinatus. The hypovascular nature of the rotator crescent may contribute to the degeneration of the rotator cuff tears (Keener et al., 2019). Ditsios et al. (2014) conducted an animal study using rat models and showed that the hypovascular tendon insertion of the atrophic chronic rotator cuff tear is the most frequent site for degeneration, fibrosis, and necrosis (Keener et al., 2019).

Age, tear size, and degeneration are known to have a negative impact on healing (Hebert-Davies et al., 2017; Keener et al., 2019; Oh et al., 2020). The healing rate was 95% for patients with age under 55 years; however, it was 43% for patients with age above 65 years (Keener et al., 2019). A tear greater than 2 cm in size had a lower healing rate (65% vs*.* 89%) and higher re-tear rate compared to a tear less than 2 cm in size (34.2% vs*.* 10.6%) (Codding and Keener, 2018; Keener et al., 2019). With the decreased muscle quality due to fatty infiltration and atrophy considered, Yamamoto et al. (2017) assumed that tears measuring 1–3 cm in length and 2–3 cm in width progress more than tears with other measurements. For high-grade, large, and massive tears, recurrent defects were found in 94% of shoulders examined (Keener et al., 2019). Between factors involved in healing impairment, a study found that age (>65 years) and tear size (13.0 mm enrollment or 9.0 mm enlargement) are correlated with degeneration (Keener et al., 2019; Ichinose et al., 2021). Degenerative changes are linked to a poor clinical outcome and tendon healing, where acute injury tears without degeneration are more likely to heal than chronic degenerative tears (Keener et al., 2019). An infiltration larger than grade 2 in the Goutallier classification represents a turning point where the healing process becomes less consistent (Hebert-Davies et al., 2017; Codding and Keener, 2018; Keener et al., 2019). Studies have demonstrated fatty infiltration as an independent risk factor for recurrent tears and defects found after repair surgery (Keener et al., 2019). Jung W. et al. (2020) conducted a study where they found that the fatty degeneration of the supraspinatus muscle and muscle atrophy of the supraspinatus and infraspinatus muscles significantly affect tear progression, demonstrating difficult spontaneous healing. In muscle atrophy, the capacity for tendon healing is decreased. Retraction of the tear, besides causing tension and improper loading, also halts the healing process (Shin, 2012; Keener et al., 2019; Tsuchiya et al., 2021).

### 2.2 Chronic rotator cuff tear and oxidative stress

Oxidative stress occurs in both acute and chronic tendon injuries. Reactive oxygen species (ROS) is significantly elevated in an injured tendon compared to a normal tendon as shown in an animal model (Lui et al., 2022). Degeneration, as observed in a chronic rotator cuff tear, has a high chance to be influenced by damage caused by ROS (Wu et al., 2011; Zheng et al., 2019; Lui et al., 2022). Oxidative stress degrades the tendon matrix because of matrix metalloproteinase-1 (MMP1) and causes metaplasia by impairing the differentiation of tendon-derived stem cells (Lui et al., 2022).

ROS is continuously produced in an injured tendon. In time, excessive ROS with an inadequate balancing antioxidant will accumulate, which leads to oxidative stress. Synovial fluid ROS and superoxide-induced oxidative stress level are known to increase in the degenerated rotator cuff and recurrent tear. Hypoxia and metabolic factors are some of the factors that can initiate oxidative stress in the tendon tissue. Inflammation cytokines present in an injured tendon are regulated by oxidative stress, but some inflammation cytokines, such as IL-β and TNF-α, further induce the production of ROS in tenocytes through nicotinamide adenine dinucleotide phosphate (NADPH) oxidase (NOX). Oxidative stress-induced inflammation also contributes to alterations in vascularization and hypoxia (Lui et al., 2022).

**FIGURE 1:**
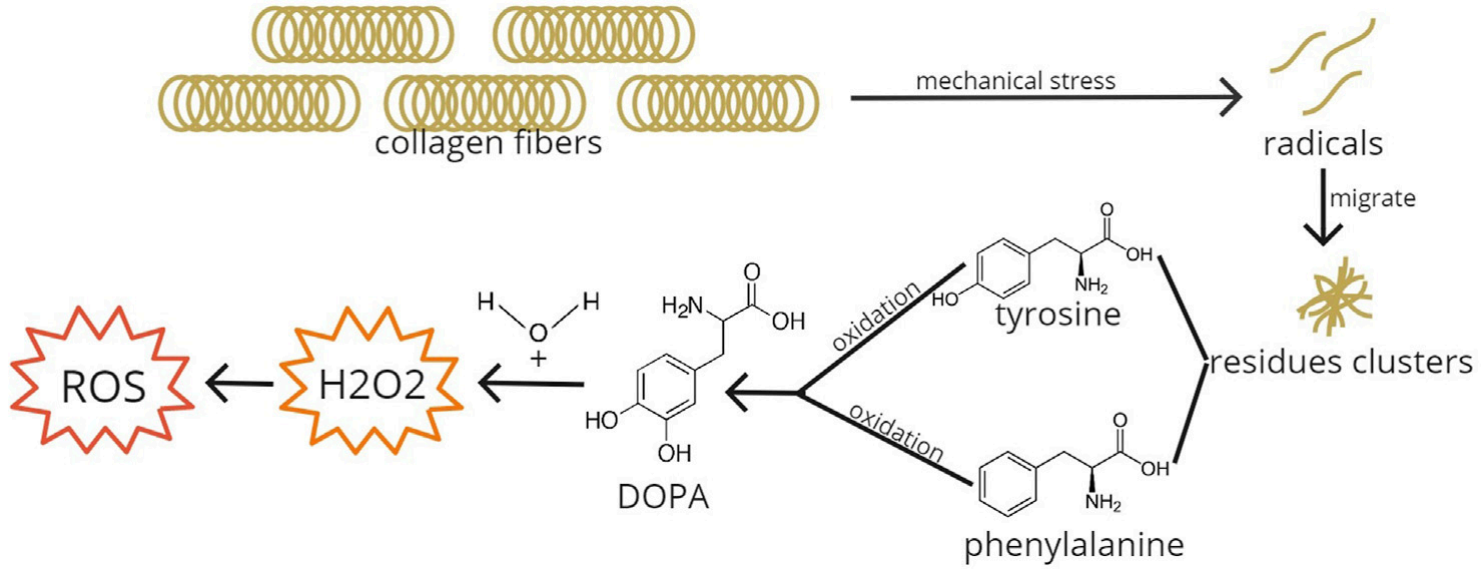
ROS formation due to mechanical stress. Collagen fibers when exposed to mechanical stress will break down into radicals and will migrate to adjacent residue clusters. In the clusters, tyrosine and phenylalanine amino acids are oxidized into DOPA. DOPA added with water transforms into H_2_O_2_, generating ROS and inducing oxidative stress. DOPA, dihydroxyphenylalanine; H_2_O_2_, hydrogen peroxide; ROS, reactive oxygen species.

Wen et al. (2016) used a rat skeletal muscle as a model for musculoskeletal injury and found that the levels of hypoxia-inducible factor (HIF) 1α and AMP-activated protein kinase (AMPK) α2, factors mediating hypoxia adaptation occurring in the injured muscle, elevated significantly at 12 h, peaked at 2 days, declined at 5 days, and reached near the normal level at 15 days. We thought that hypoxia plays an important role in the occurring pathways as a response to rotator cuff injury. The chronic overloading of the enthesis in PTRCTs and hypovascular nature of the proximal tendon insertion, different from other areas of tendons or muscles, will lead to prolonged hypoxia (Ditsios et al., 2014; Keener et al., 2019). In the hypoxic state, xanthine oxidase and NADPH will be activated and ROS production will occur, resulting in oxidative stress (Ji and Yeo, 2019). Tendon overloading generates cellular stress in tendon cells (Lui et al., 2022). Regulation of genes related to ROS is highly influenced by mechanical loading. The plasma total oxidant status is elevated in tendon overuse (Lui et al., 2022). Tendons are collagen-dense connective tissue that allows human body movements and serves as a force bridge between muscles and bones (Montagna et al., 2022). Collagen affected by mechanical stress would generate radicals that will migrate to the adjacent residue clusters and would settle as dihydroxyphenylalanine (DOPA), the oxidized form of tyrosine or phenylalanine residues. In the presence of water, DOPA would transform into hydrogen peroxide (H_2_O_2_) (Figure 1) (Lui et al., 2022). Superoxide anions produced by oxidation are mainly dismuted into H_2_O_2_ by superoxide dismutase (SOD) in the mitochondria, the main source of ATP production by oxidative phosphorylation (OXPHOS), which generates ROS (Chevallier et al., 2020; Kim et al., 2020). Although the formation of DOPA and H_2_O_2_ with scavenged radicals in collagen is considered a protective mechanism against oxidative stress, this oxidative stress inducer would cause oxidative damage, endoplasmic reticulum (ER) stress activation, tendon cell apoptosis, and matrix degradation through the induction of MMP1 mRNA expression if there is an excessive production of H_2_O_2_ (Lui et al., 2022). Through these mechanisms, oxidative stress seems to be occurring in rotator cuff tears.

### 2.3 Oxidative stress and autophagy

ROS accumulation would induce autophagy as the regulator of cell oxidation (Gao, 2019). Oxidative stress in the enthesis leads to cell apoptosis, fibrosis, and degeneration. The expression of antioxidants, such as peroxiredoxin 5 in fibroblast and endothelial cells in tendons, provides protection from apoptosis and cell function loss. Oxidative stress may induce apoptosis and tendon matrix degradation. Antioxidant deficiency in mice shows enthesis degeneration, which supports the involvement of oxidative stress in tendon degeneration (Lui et al., 2022). Mitochondria accommodate changes caused by imbalanced ROS and antioxidants by mitophagy (Ji and Yeo, 2019). With hypoxia, a factor that could initiate oxidative stress, autophagy flux increases. Chen et al. (2017) conducted a study on mouse C2C12 myotube cells treated with cobalt chloride to induce autophagy. They found a significant increase in LC3II, a marker for autophagosomes, and a decrease in p62, an autophagic adapter protein degraded during increased autophagy (Song et al., 2012; Chen et al., 2017; Runwal et al., 2019). The elevated autophagic activity could be initiated excessively in rotator cuff tears, leading to tissue destruction, which may be pathologic. Autophagy could be initiated by HIF-1, the mammalian target of rapamycin (mTOR), and UPR mechanisms (Fang et al., 2015).

### 2.4 HIF-1-mediated autophagy

**FIGURE 2:**
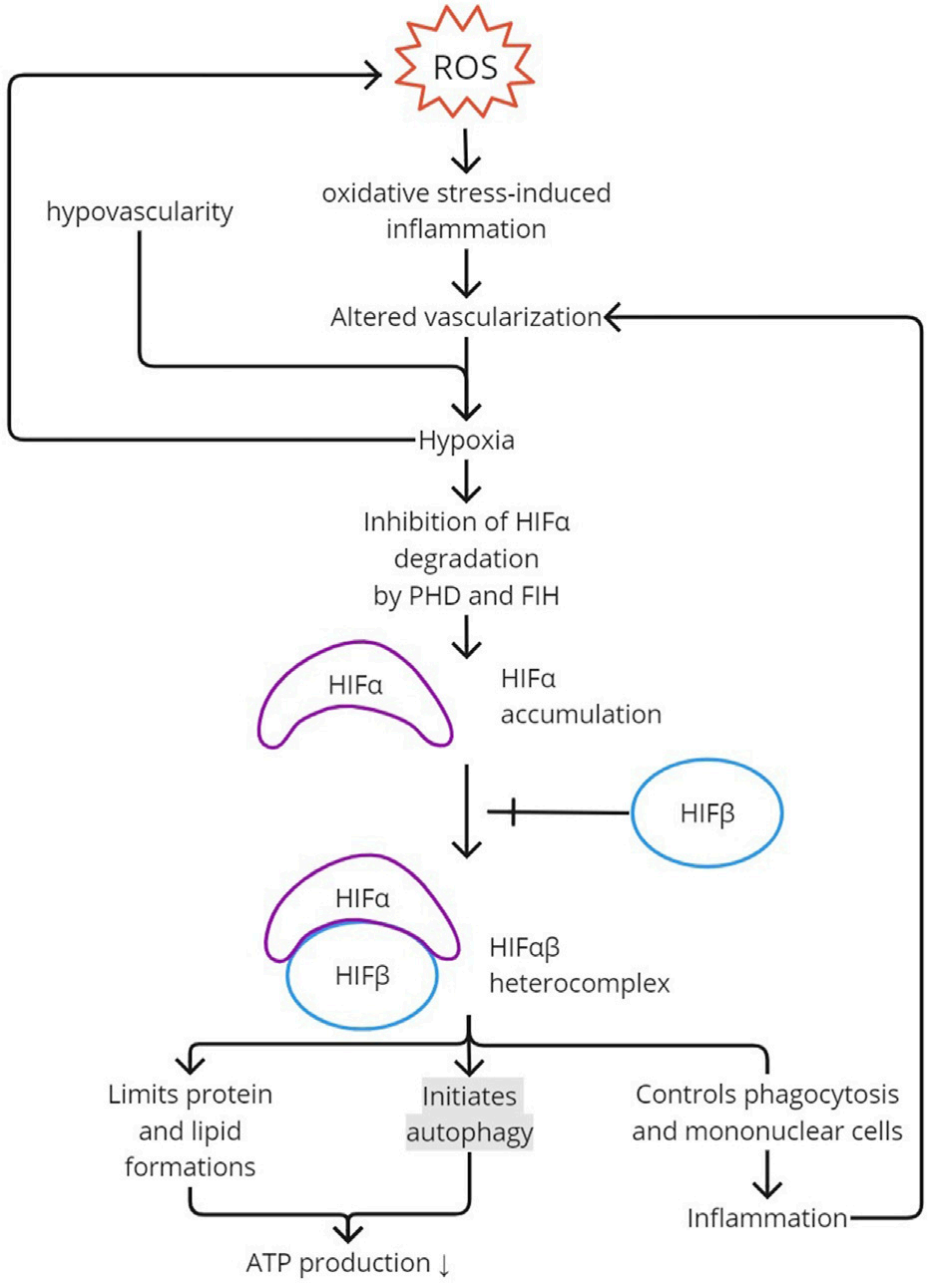
HIF activation. ROS creates oxidative stress and induces inflammation. Inflammation would alter vascularization and, together with the hypovascularity of the enthesis, would create hypoxia. Hypoxia inhibits PHD and FIH activity in degrading HIFα. The accumulation of HIFα added with HIFβ creates an HIFαβ heterocomplex, the activated form of HIF. To limit ATP production and, therefore, ROS production, autophagy is initiated with a limitation in protein and lipid formation. Activated HIF also contributes to inflammation by regulating phagocytosis and mononuclear cells. ROS, reactive oxygen species; PHD, proline hydroxylase; FIH, factor-inhibiting HIF; HIF, hypoxia-inducible factor.

HIFs with an α-subunit in normoxia is inhibited by proline hydroxylase (PHD), leading to post-translational modification and proteasomal degradation (Song et al., 2012; Bartoszewska and Collawn, 2020). The subunit is also degraded by pVHL through ubiquitinylation (Song et al., 2012). At the transcription stage, it is inhibited by factor-inhibiting HIF (FIH). During a hypoxic state, post-translational modification is inhibited, resulting in an α-subunit and transcriptionally active heterocomplex HIF-αβ accumulation (Figure 2). The activated HIF decreases ROS production. The activated mechanisms are decreased protein production, mitophagy, and autophagy to minimize ATP needs, which utilizes oxidative phosphorylation rather than a nonoxidative mechanism in hypoxic-state ATP production (Bartoszewska and Collawn, 2020). HIF-1α also regulates phagocytosis and mononuclear cells, which are important in inflammation (Song et al., 2012).

**FIGURE 3:**
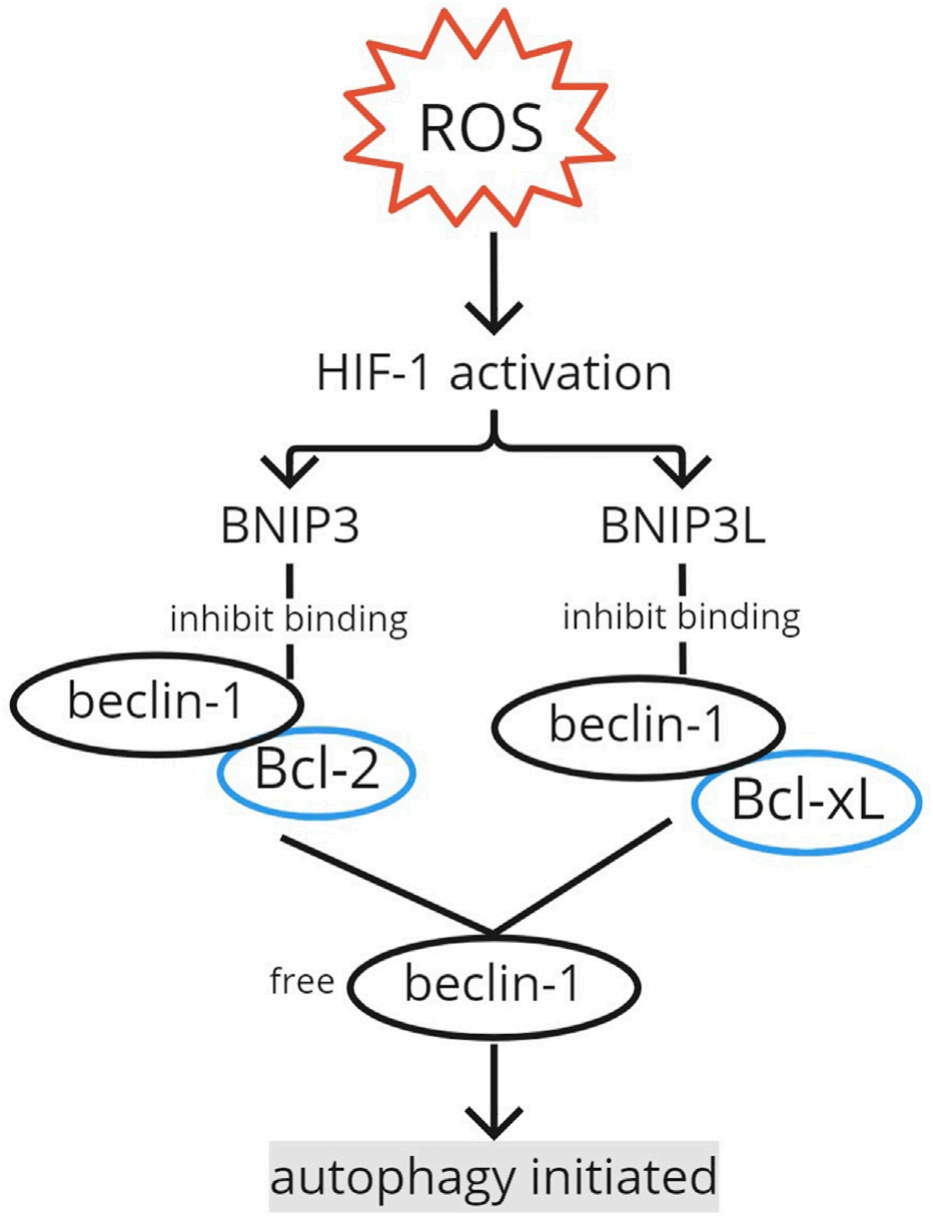
Autophagy initiation by HIF-1. The activation of HIF-1 by ROS induces BNIP3 and BNIP3L transcription. BNIP3 and BNIP3L interact with Bcl-2 and Bcl-xL, respectively, inhibiting their binding with beclin-1. The free beclin-1 initiates the autophagy mechanism. ROS, reactive oxygen species; HIF, hypoxia-inducible factor; BNIP, Bcl-2 adenovirus E1B 19-kDa interacting protein.

The autophagy mechanism is induced in hypoxia (Chen et al., 2017; Wang et al., 2018). Wang et al. found that in the musculoskeletal field, beclin-1, involved in autophagy initiation, increases during hypoxia in stem cells derived from rat genioglossus muscles. Autophagosome vacuoles with cytoplasmic organelles and vesicles inside were observed to be significantly increased in hypoxia. The induction of autophagy in hypoxia mostly involves the HIF-1α/Bcl-2 adenovirus E1B 19-kDa interacting protein (BNIP) 3 signaling pathway (Figure 3). Hypoxia-induced autophagy was thought to have removed mitochondria for ROS over-accumulation prevention, but it is now known to cause autophagic cell death (Zhang et al., 2018). Chen et al. (2017) also found that HIF-1α, BNIP3, and beclin-1 are upregulated in hypoxia-induced autophagy. Similar results for HIF-1α and BNIP3 were found in Song et al. (2012) in human periodontal ligament cells. HIF-1 initiates the transcription of BNIP3 and BNIP3L, which interact with Bcl-2 and Bcl-xL, inhibiting their bond with Vps34 and releasing beclin-1, resulting in autophagy induction (Song et al., 2012; Chen et al., 2017; Zhang et al., 2018). Therefore, besides its role in maintaining homeostasis, HIF-1 can potentially over-activate autophagy through beclin-1.

### 2.5 mTOR pathway-mediated autophagy

**FIGURE 4:**
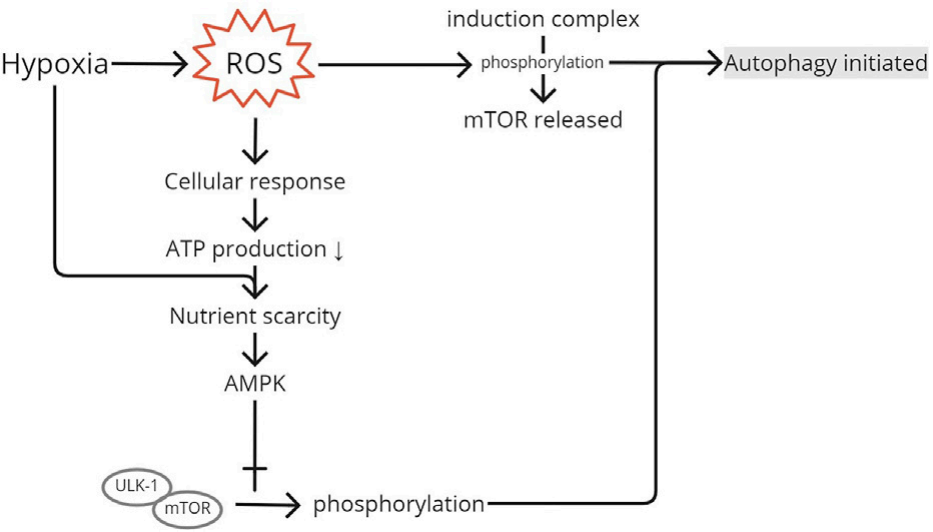
mTOR pathway activation. ROS produced after the injury and produced because of hypoxia will generate a cellular response to produce less ATP to limit ROS as its residual product. Low ATP and hypoxia create nutrient scarcity in the cell and initiate AMPK to interact with the ULK1 complex, causing phosphorylation. Phosphorylation and separation of mTOR from the induction complex initiate autophagy. ROS, reactive oxygen species; AMPK, AMP-activated protein kinase; ULK1, unc-51-like autophagy-activating kinase 1; mTOR, mammalian target of rapamycin.

Oxidative stress removes mTOR from the induction complex, causing phosphorylation (Figure 4). The activation of the mTOR pathway by phosphorylation initiates autophagy. Xu et al. (2020) found an elevation of LC3 in the activation of the mTOR pathway. Energy production is limited in the hypoxic state to retain ROS production (Bartoszewska and Collawn, 2020). Nutrient scarcity would initiate AMPK to interact with the unc-51-like autophagy-activating kinase 1 (ULK1) complex. The interaction would cause phosphorylation and mTOR separation. The dissociation of mTOR from the ULK1 complex enables ULK1 to trigger the translation of autophagosomes (Chen et al., 2017). Therefore, the phosphorylation of the complex downregulates mTOR, as shown in Zheng et al., where the injection of an mTOR inhibitor to rat supraspinatus results in increased amounts of autophagosomes and autophagolysosomes. Chen et al. (2017) also measured the levels of p-mTOR/mTOR and p-AMPKα/AMPKα and found their decreased and increased levels, respectively, in hypoxia-induced autophagy, confirming their roles in autophagy.

Oxidative stress and overloading activate mTOR complex 1 (mTORC1). The signaling pathway of mTOR when mTOR is downregulated is associated with metaplasia, fatty infiltration, and muscle atrophy (Ji and Yeo, 2019; Lui et al., 2022). The mTOR pathway reduces protein synthesis and induces autophagy, similar to that of HIF (Bartoszewska and Collawn, 2020). Conversely, Xie et al. (2019) used *Caenorhabditis elegans* and found that the signaling of mTORC1 negatively affects eukaryotic elongation factor 2 kinase (eEF2K), causing reduced inactivation by the phosphorylation of eEF2 needed for ribosome movement along the mRNA, which enhances the accuracy of protein synthesis, instead of affecting the quantity of protein production.

### 2.6 UPR-mediated autophagy

The cellular response during hypoxia maintains survival under the unstable condition and restores oxygen homeostasis. A metabolic switch happens because of HIF activation, and other cellular responses would eventually disrupt cellular homeostasis. ATP limitation, as mentioned in the previous section, reduces protein and lipid synthesis, disturbs protein folding in the formation of a disulfide bond, and increases ROS (Bartoszewska and Collawn, 2020).

ROS from its main source, mitochondria, enters the ER through mitochondrial-associated membranes or diffuses as a part of redox homeostasis. Redox modifications of autophagy components have been reported to affect the induction of autophagy, alter ER redox homeostasis, and initiate autophagy. In the ER itself, NOX4 in the ER membrane acts as a catalyst of ROS production. Oxidative protein folding is one of the sources of H_2_O_2_ in the ER (Zhang et al., 2019). The overproduction of H_2_O_2_, an oxidative stress inducer involved in a chronic rotator cuff tear, would cause ER stress (Lui et al., 2022). Some proteins are expressed higher in a rotator cuff tear. A few of those proteins are matrix-degrading enzymes, pro-MMP1 protein, a protein involved in inflammatory response (S100A11), a protein needed for lipid droplet formation (PLIN4), and a protective ER protein produced in the hypoxic state (HYOU1) (Lui et al., 2022). Intracellular homeostasis alterations and extracellular stimuli cause protein misfolding (Han et al., 2013). ROS would affect the redox environment, which disrupts the proteins that enter ER for proper protein folding. Under normal conditions, the misfolded proteins are degraded via the ubiquitin–proteasome system (UPS) (Zeeshan et al., 2016). ROS with ER stress aggregates small protein and impair the UPS, thus needing autophagy activation for misfolded protein elimination (Zeeshan et al., 2016; Hetz et al., 2020). An elevation in protein secretion or protein folding disruption would create ER stress (Hetz et al., 2020). Protein folding and maturation are processed in the ER, assisted by compartment-specific molecular chaperones. Proteostasis (protein homeostasis) is maintained by cells by adjusting the complement of chaperones to match the amount of protein synthesized. Failure in the chaperones to balance the protein synthesis load will cause excess non-native ER clients (ER stress), causing proteotoxic misfolding and aggregate formation (Preissler and Ron, 2019; Hetz et al., 2020). The increase in protein synthesis would cause a necessary elevation of disulfide bond formation in the ER, involving electron shuttle through ER oxidoreductin (ERO) 1α oxidation, exchanging the disulfide bond with protein disulfide isomerase (PDI) where H_2_O_2_ is generated, causing further ER stress, oxidative stress, protein oxidation, and overloaded misfolded protein, leading to UPR activation (Han et al., 2013; Chevallier et al., 2020). Calcium signaling by ERO1α induces oxidative stress, especially in the mitochondria, and ROS production (Chevallier et al., 2020). A cycle of pathway can be occurring from the increased protein synthesis in response to rotator cuff injury combined with oxidative stress, causing ER stress, which leads to protein misfolding that activates UPR and results in further oxidative stress.

**FIGURE 5:**
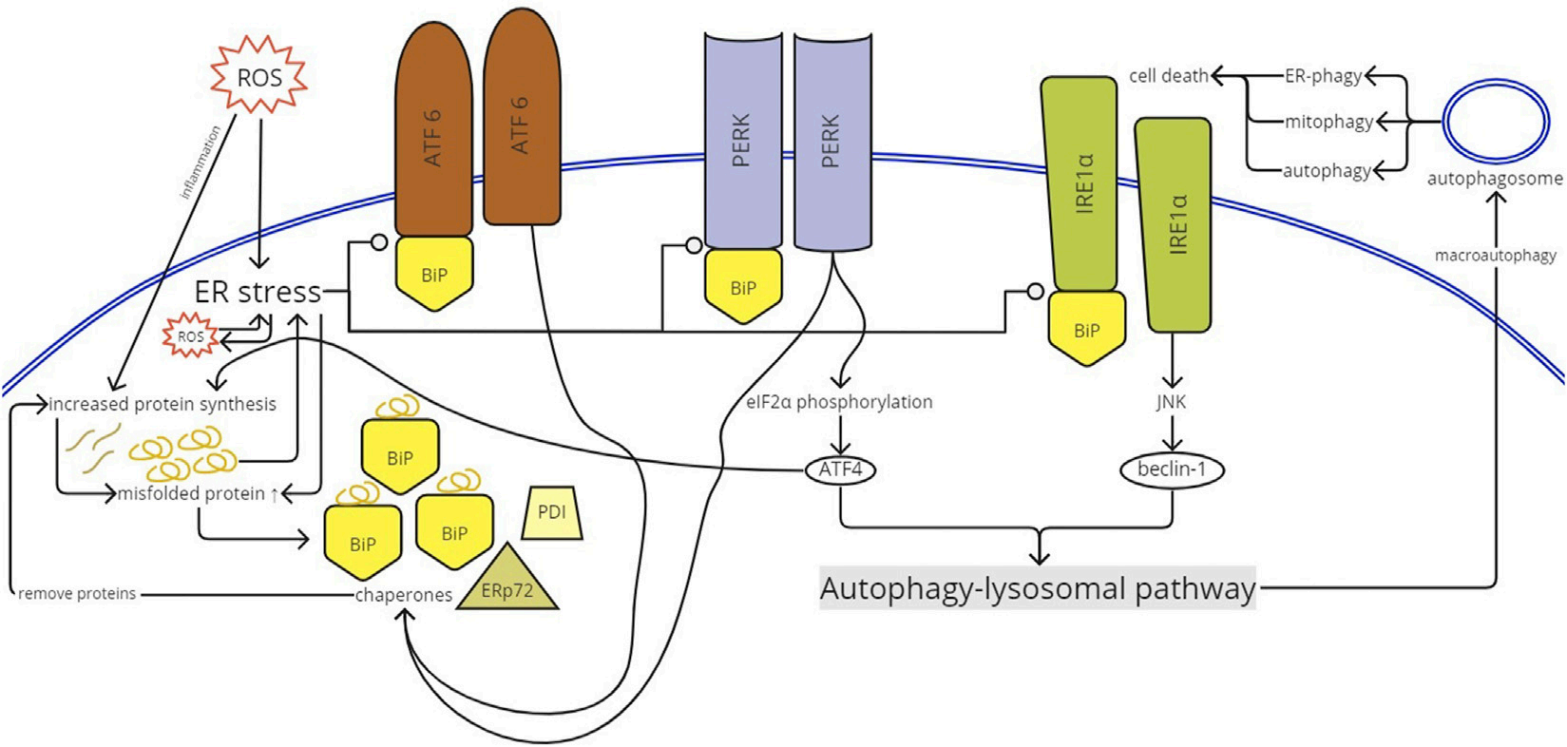
UPR activation. ROS enters the ER and causes ER stress. ER stress would create ROS inside the ER and *vice versa*. Inflammation induced by ROS increases protein synthesis to produce proinflammatory proteins. ER stress releases BiP from the luminal domain of the stress sensors (PERK, IRE1α, and ATF6). Once activated, ATF6 and PERK stimulate chaperone (BiP, ERp72, and PDI) expression. The chaperones will remove proteins, further inducing protein synthesis. The increased protein synthesis causes impairment in protein folding and produces misfolded proteins. The misfolded proteins create more ER stress and *vice versa*. The activated PERK phosphorylated eIF2α that supports ATF4 translation. ATF4 mediates and, therefore, increases protein synthesis. IRE1α activation results in the initiation of IRE1α-JUN N-terminal kinase (JNK) signaling, which activates beclin-1. ATF4 and beclin-1 trigger the autophagy-lysosomal pathway, creating autophagosomes, causing autophagy and even cell death. ROS, reactive oxygen species; ER, endoplasmic reticulum; BiP, binding immunoglobulin protein; PERK, protein kinase RNA-like ER kinase; IRE1α, inositol-requiring protein; ATF, activating transcription factor; PDI, protein disulfide isomerase; JNK, JUN N-terminal kinase.

ER stress would activate a series of adaptive mechanisms called UPR to cope with the alterations of protein folding (Figure 5). Information regarding the protein folding status is delivered to the nucleus and cytosol via UPR. UPR in vertebrates is a complex signaling pathway with multiple cellular responses mediated by the following three known major stress sensors: inositol-requiring protein 1 (IRE1) α and β, ATF6 α and β, and protein kinase RNA-like ER kinase (PERK) (Hetz, 2012; Preissler and Ron, 2019; Bartoszewska and Collawn, 2020). The stress-sensing luminal domain (LD) of inactivated IRE1 is bound with binding immunoglobulin protein (BiP), an ER quality control molecule and chaperone. BiP in ER stress would release itself from the LD bond, activating UPR, and bind with unfolded proteins to regulate folding and degradation (Preissler and Ron, 2019). Although demonstrated in β-cells in Sowers et al. (2018), PERK and ATF6 activated in ER stress would stimulate chaperone expression such as BiP, protein disulfide isomerase (PDI), and ERp72. The activation of the response would lead to protein synthesis attenuation through translation inhibition, mRNA decay, and autophagy. If ER stress becomes irreversible, the pathway removes damaged cells by apoptosis (Hetz, 2012; Preissler and Ron, 2019; Bartoszewska and Collawn, 2020).

Macroautophagy is one of the responses of the UPR pathway to eliminate unfolded proteins (Han et al., 2013; Smith, 2018; Hetz et al., 2020). This bulk degradation mechanism involving the lysosomal pathway eliminates aggregation of abnormal protein and damaged ER (Han et al., 2013). The mechanism is signaled by IRE1α and PERK. PERK activated by ER stress through dimerization, oligomerization, and autophosphorylation results in eukaryotic translation initiator factor 2α (eIF2α) phosphorylation, which translates mRNA encoding ATF4. ATF4 controls the prosurvival gene level related to autophagy by binding to the proximal promoter region of its target genes (Hetz, 2012; Han et al., 2013). In addition, ATF4 target genes include *Atf2*, *Gadd34*, *Trib3*, *Wars*, and aminoacyl tRNA synthetases. The target gene *Gadd34/Ppp1r15a* regulates protein phosphatase 1 (PP1), causing feedback for eIF2α dephosphorylation. However, most ATF4 target genes are involved in protein synthesis (Han et al., 2013; Hetz et al., 2020). Protein synthesis mediated by ATF4 is enhanced by CHOP, expressed because of eIF2α phosphorylation and ATF6f induction (Han et al., 2013; Bartoszewska and Collawn, 2020). The activation of ATF4 and CHOP further increases protein synthesis. In the uncorrected ER stress, these proteins would be misfolded (Han et al., 2013). ATF6 that regulates transcription of ER chaperones and enzymes promoting ER protein regulation is ATF6P50, the fragment released from full length ATF6p90 by cleavage in site-1 protease (S1P) and site-2-protease (S2P) to release basic leucine zipper (bZIP) transcription factor-containing fragments (Hetz et al., 2020).

IRE1α is originally repressed by binding to BiP (GRP78 or HSPA5). During ER stress, the BiP bond with IRE1α is separated because of its preferential binding with the misfolded protein, allowing IRE1α phosphorylation, dimerization, and cluster formation. High stress would enlarge the cluster, but prolonged ER stress would result in cluster dissociation and attenuated activity. Through ER stress-activated IRE1α, the adapter protein TNFR-associated factor 2 (TRAF2) is recruited (Hetz, 2012). JUN N-terminal kinase (JNK) is one of the targets of TRAF2, mediating IRE1α-JNK signaling. The signaling pathway would activate beclin-1 (autophagy regulator) and would trigger autophagy (Hetz, 2012; Smith, 2018). IRE1α binds to protein receptor optineurin, which functions in activating autophagy for mitochondria (mitophagy) observed in neuron cells and controlling the stability of IRE1α (Wong and Holzbaur, 2014; Hetz et al., 2020).

### 2.7 Oxidative stress-induced autophagy in a chronic rotator cuff tear

**FIGURE 6:**
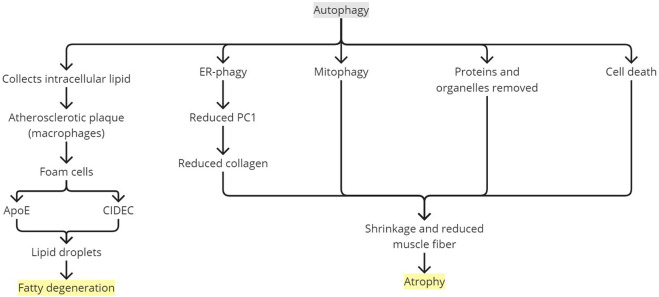
Rotator cuff degeneration by autophagy. ApoE, apolipoprotein E; ER, endoplasmic reticulum; PC1, type 1 procollagen.

An over-elevated ROS level, as observed in chronic rotator cuff injuries, could result in uncontrolled and unregulated autophagy (Chen et al., 2017). Hirunsai and Srikuea (2021) found increased LC3I conversion to LC3II and p62 protein expression in rat soleus and plantaris muscles 8 and 14 days after tenotomy. Autophagy under normal conditions removes or recycles proteins and organelles to maintain homeostasis at the cellular level (Chen et al., 2017). The cellular defense mechanism against oxidative stress is available through the autophagy-lysosomal pathway. Normally, the autophagy mechanism selectively removes damaged proteins and organelles (Chen et al., 2017; Lui et al., 2022). Autophagy is “self-eating,” a normal mechanism of tissue repair or conversion to energy needed for the repair (Wang et al., 2018). Autophagy helps in balancing synthesis and degradation (Chen et al., 2017). However, excessive mechanisms will degenerate the tendon, resulting in an abnormal state (Figure 6) (Wu et al., 2011; Song et al., 2012; Joshi et al., 2014; Hirunsai and Srikuea, 2021). The imbalance and overstimulated, therefore unregulated, autophagy is increased under a catabolic condition, as observed in the hypoxic enthesis tear where energy production is switched to glycolysis due to ROS and HIF-1α (Calejo et al., 2021; Hirunsai and Srikuea, 2021). Hirunsai and Srikuea (2021) applied heat stress to their subjects to create an anabolic condition and confirmed the presence of an imbalanced autophagy-lysosomal pathway.

Chronicity complicates the rotator cuff tear, mostly manifesting as muscle atrophy (Zheng et al., 2019). The loss of skeletal mass or muscle wasting has been reported to involve autophagy (Joshi et al., 2014; Chen et al., 2017). Currently, among all members of the autophagy-lysosome system, macroautophagy is the only member known to be involved in muscle atrophy (Ji and Yeo, 2019). Enhanced protein degradation and diminished protein synthesis are the hallmarks of atrophy (Zheng et al., 2019). The autophagy mechanism is fundamental to several tissues to control degradation and recycle cellular components engulfed in autophagosome vesicles. It is an early cellular response to stress. Selective ER autophagy (ER-phagy) controls type 1 procollagen (PC1) secretion, a precursor for type 1 collagen (COL1), the major component of the tendon extracellular matrix. Montagna et al. (2022) conducted a study using human gracilis tendons and murine Achilles tendons and showed that the PC1 level is reduced in the presence of autophagosomes and increased when inhibition is performed. With misfolded PC1 aggregation, the rise and fall of the PC1 level affected by autophagosome presence were even more prominent. These indicated that autophagy eliminates PC1, especially if it was misfolded. Tendon tissue with autophagy induction showed downregulated expression of the *COL1A1* gene, irregular collagen fibrils, and significantly reduced mechanical strength (both in maximum stress and strain). Muscle atrophy is known to have tissue fibrosis and cellular apoptosis involvements (Jung et al., 2020a; Hirunsai and Srikuea, 2021). Autophagy may cause cell death and may trigger apoptosis, resulting in autophagy-mediated cell death (ACD) (Jung et al., 2020b). Wu et al. (2011) showed increased ACD in a chronic rotator cuff tendon tear with histologically moderate changes (grade 2) when compared to a normal tendon (grade 0) and a tendon with slight changes (grade 1). However, in a tendon with the total loss of fiber structure (grade 3), ACD was decreased compared with a tendon with moderate changes. Meanwhile, myofibroblast percentage was elevated with the worse tendon tissue changes as an attempt to revive tendon integrity. With persistent overload and malnutrition, as in a chronic condition and hypoxia, ACD may surpass cell migration and proliferation, causing cell scarcity and reduction in cell density (Wu et al., 2011). Protein elimination, organelle removal, and ACD would shrink and reduce muscle fibers, resulting in atrophy (Wu et al., 2011; Bonaldo and Sandri, 2013; Hirunsai and Srikuea, 2021). This has been confirmed in Joshi et al. (2014) by finding the upregulation of LC3II with autophagy as the major degradation process in an atrophic rat supraspinatus model.

Inflammation initiated by ROS and HIF-1α could directly lead to structural changes in tendons (Song et al., 2012; Lui et al., 2022). Proinflammatory cytokines, such as IL-1β and MMP-8 (collagenase 2), play roles in tissue destruction. IL-1β induces fibroblasts to produce collagenase and stromelysin or other destructive metalloproteinases (Song et al., 2012). Collagenase 2 degrades COL1, the major component of the enthesis (Song et al., 2012; Montagna et al., 2022). Inflammation helps in developing fatty infiltration and muscle atrophy post injury in a rotator cuff, leading to tear worsening. Anti-inflammation treatment is known to reduce fatty infiltration and fibrosis, improving muscle strength up to twofold in load to failure, and increase healing in the repaired enthesis, as observed in studies using animal models. Autophagy activity has been observed in a degenerated tendon, causing fatty infiltration (Gumucio et al., 2012; Oak et al., 2014). Gumucio et al. (2012) demonstrated the expression of Vps34 and beclin-1 in torn rat rotator cuffs with lipid accumulations, suggesting the involvement of autophagocytic pathways in the chronic rotator cuff tear. Autophagy collects intracellular lipid in atherosclerotic plaque regulated by macrophages. The macrophages then differentiate into foam cells expressing apolipoprotein E (ApoE), a major component of very-low-density lipoproteins and chylomicron particles, and CIDEC, a cell death-inducing DFF45-like effector (CIDE) protein (Gumucio et al., 2012; Gao et al., 2017). Both ApoE and CIDEC form lipid droplets involved in fat metabolism (Gumucio et al., 2012).

**FIGURE 7:**
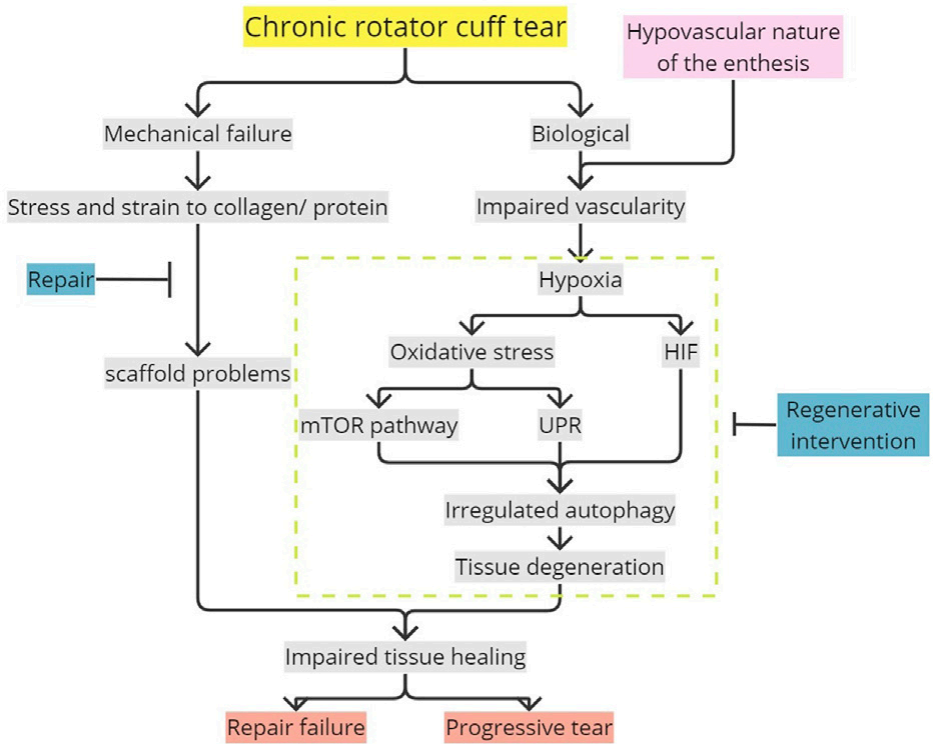
Potential mechanism in impaired enthesis tissue healing mediated by oxidative stress-induced autophagy. Flat-ended line, intervention; mTOR, mammalian target of rapamycin; UPR, unfolded protein response; HIF, hypoxia-inducible factor.

Treatments such as rotator cuff repair and postoperative rehabilitations are performed to anatomically and mechanically return the torn rotator cuff into its original state, reducing further strain and stress to allow tissue healing (Sgroi and Cilenti, 2018). In the enthesis, regenerative engineering by administering local biological therapeutics has been cultivated using biodegradable synthetic polymers as a potential treatment to improve postoperative enthesis-healing quality (Prabhath et al., 2021). With this review, it is revealed that ROS plays an important role in activating excessive autophagy in the torn rotator cuff, which causes tissue damage and degeneration, restricting proper healing. Detailed research can be conducted to further confirm the autophagy-related mechanisms occurring in the torn rotator cuff. Biological interventions reducing ROS formation, oxidative stress, and autophagic activity could be considered to allow better tissue healing in the rotator cuff tear (Figure 7).

## 3 Conclusion

Oxidative stress occurring in a hypovascularized chronic rotator cuff tear due to hypoxia and ROS accumulation would result in unregulated autophagy directly or autophagy mediated by HIF-1, mTOR, and UPR. These mechanisms, leading to autophagy in the enthesis if occurring chronically, would cause degenerative changes such as denervation degeneration and fatty degeneration. These would lead to disrupted enthesis healing and tear progression, complicating repair with a high risk of re-tear. The cascades explained were a potential contributor to the high recurrent rate of the rotator cuff enthesis tear. Unregulated excessive autophagy caused by oxidative stress, which results in tissue degeneration and potentially produces pathological tissue, should be accounted for future studies regarding rotator cuff tears. Therefore, further research is required for better understanding, and biological interventions can be considered in addition to surgical repair to enhance tissue healing.
